# Prevalence and risk factors of work-related musculoskeletal disorders among older hospital cleaners in Wenzhou, China

**DOI:** 10.3389/fpubh.2025.1727574

**Published:** 2025-11-26

**Authors:** Wei Zhou, Yiyu Chen

**Affiliations:** Department of Art, Southeast University, Nanjing, Jiangsu, China

**Keywords:** work-related musculoskeletal disorders, hospital cleaners, older workers, occupational health, delayed retirement policy

## Abstract

**Introduction:**

Work-related musculoskeletal disorders (WMSDs) are highly prevalent among hospital cleaners globally, yet epidemiological data among older hospital cleaners in China remains limited in the context of workforce aging and delayed retirement policies.

**Methods:**

This cross-sectional study was conducted among hospital cleaners in three tertiary public hospitals in Wenzhou, China. Data were collected using the Chinese version of the Standardized Nordic Musculoskeletal Questionnaire (C-NMQ). A total of 246 hospital cleaners aged ≥50 years and 80 cleaners aged <50 years (as an age comparison group) were included. Chi-square tests and Cochran-Armitage trend tests were used to analyze age-related prevalence patterns. Multivariable logistic regression with backward stepwise selection was employed to identify independent risk factors.

**Results:**

The 12-month prevalence of WMSDs among cleaners aged ≥50 years was 32.1%. The most commonly affected body regions were the shoulder, knee, and neck. Multivariable analysis revealed that working in uncomfortable postures (AOR = 2.87) and staff shortage (AOR = 2.71) were independent risk factors for WMSDs. Sensitivity analysis showed that working >50 h per week significantly increased the risk of WMSDs.

**Discussion:**

Older hospital cleaners experience a considerable burden of WMSDs. Working in uncomfortable postures, staff shortage, and long working hours are major risk factors. Comprehensive interventions targeting ergonomic improvements, organizational management optimization, and occupational health surveillance are recommended, with priority given to early screening and intervention, particularly for workers transitioning into older age groups. These findings provide evidence for occupational health protection in the context of the delayed retirement policy in China.

## 1 Introduction

The demographic shift and global aging workforce have prompted governments to adjust their retirement policies, raising labor market participation among older individuals (1–3). These policies have led to a growing number of older adults returning to work (4–6). The Organization for Economic Cooperation and Development (OECD) reported that the labor force participation rate of individuals aged 55 years and older in OECD countries reached 83.5% in 2024 (7). Due to limitations in skills, educational attainment, and health status, many older workers are compelled to engage in low-skilled and physically demanding jobs, such as hospital cleaning (8–12). Many studies indicate significant aging characteristics in this occupational group (13, 14). A study in Texas showed that 54.7% of hospital cleaners were aged 39–58 years and 19.8% were aged ≥59 years (15); a Brazilian study reported that 34.9% were aged 49–62 years (16). These older workers are more vulnerable to severe health damage due to the cumulative effects of aging and sustained workplace hazards (8).

Among these workplace hazards, work-related musculoskeletal disorders (WMSDs) emerge as the most critical concern (17, 18). Globally, the prevalence of WMSDs among hospital cleaners ranges from 51 to 81.9%, substantially higher than that in the general occupational population. Commonly affected body regions include the shoulders, back, knees, and neck (16, 19–22). WMSDs refer to injuries or functional impairments of muscles, bones, and joints caused by occupational activities (23), constituting a major cause of disability, absenteeism, and long-term sick leave (24–26). This exacerbates economic vulnerability in this population, increases employers’ labor costs, and places additional strain on already stressed social security systems (3, 27–32). This elevated risk stems from the unique characteristics of hospital cleaning work, which creates high WMSDs exposure through multiple pathways: (1) Hospital cleaning plays a crucial role in reducing pathogen cross-contamination (10, 33, 34). The cleaning standards are stringent and work intensity is high, with high-risk areas (such as operating rooms and intensive care units) requiring cleaning every 4 h (35); (2) working hours are long and the 24-h shift systems result in prolonged exposure to occupational hazards (25); (3) maintaining awkward postures or bearing high joint loads increases WMSDs risk (15, 36).

Beyond these workplace exposure factors, the relationship between age and WMSDs remains particularly controversial. Some studies reported that WMSDs prevalence among cleaners increases with age, peaks at 35–45 years, and then declines (30). A Texas study reported similar conclusions, reporting prevalence rates of 68.96% among those aged 39–58 years and 66.66% among those aged ≥59 years (15). However, the Global Burden of Disease study revealed significant increases in both WMSD case numbers and prevalence in the 50–59 age group (37). Many studies also reported that those aged ≥50 years have a higher prevalence of WMSDs (33). This paradox may stem from the healthy worker effect, whereby workers unable to tolerate disease symptoms exit the labor market during middle age (38). A study of middle-aged workers recommended enhanced WMSDs screening during the 35–50 age window, as pain symptoms begin to emerge during this period but workers have not yet left their positions (39).

Hospital cleaners aged ≥50 years who remain employed constitute a unique population. They have undergone health selection while facing age-related physiological decline. Their WMSDs patterns and risk factors may differ fundamentally from those of younger workers. Furthermore, WMSDs often have a latency period of several months to years between symptom onset and clinical diagnosis (40). Combined with older workers’ higher pain tolerance and economic dependence on work, this may result in a large underrecognized population with symptomatic but untreated WMSDs. Therefore, epidemiological investigations specifically targeting hospital cleaners aged ≥50 years who remain employed are urgently needed.

Despite the growing recognition of WMSDs as a major occupational health concern, the evidence base remains disproportionately concentrated in high-income settings (41–43). Research from low- and middle-income countries (LMICs) is notably limited, creating challenges for policymakers in these regions who must make decisions about aging workforces without adequate contextual evidence (44). Given marked differences in labor regulations, occupational health infrastructure, and socioeconomic conditions across countries, findings from high-income regions may not be directly generalizable to LMIC contexts (44). This necessitates locally grounded research to inform retirement policies and occupational health interventions in these settings. Among LMICs, this imperative is especially pressing in China, which faces unique challenges as one of the world’s fastest-aging countries (44, 45). An epidemiological study in China from 2018 to 2020 reported a WMSD prevalence of 54.2% among healthcare workers (30). Many studies focus on emergency medical service workers (46) and medical personnel (47, 48). However, older workers who constitute the majority of hospital cleaning staff have not received sufficient attention in China. Moreover, widespread outsourcing of hospital cleaning services to private companies has created minimal occupational health surveillance, leaving older hospital cleaners in China particularly vulnerable to inadequate workplace protections and limited access to preventive interventions (44). Additionally, due to lower educational attainment, they often lack adequate knowledge and skills regarding work-related health threats and workplace hazards (10, 49). Considering the health impairments experienced by older adults are often irreversible, timely identification and intervention are essential to prevent progression to irreversible disability. However, empirical evidence on the WMSDs prevalence, risk factors, and prevention needs among hospital cleaners aged ≥50 years in China remains critically lacking, impeding the development of targeted interventions and evidence-based retirement policies for this vulnerable population.

Thus, three fundamental questions need to be addressed: (1) What is the prevalence of WMSDs among hospital cleaners aged ≥50 years in China? (2) Which risk factors are associated with WMSDs in this population? (3) What evidence-based prevention strategies can be tailored to address the unique vulnerabilities of older workers in this occupation? Answering these questions is essential for informing occupational health policy and practice in the context of workforce aging and delayed retirement.

To address these critical questions, we conducted a cross-sectional study in Wenzhou. Wenzhou is a coastal city in eastern China with a well-developed healthcare system. Hospital cleaning service outsourcing is widespread in Wenzhou, representing a typical employment model for hospital cleaners in Chinese urban public hospitals. Focusing on hospital cleaners aged ≥50 years in Wenzhou, this study aimed to: (1) describe the prevalence of WMSDs in this population; (2) identify risk factors associated with WMSDs; and (3) propose evidence-based prevention and intervention strategies for this population.

## 2 Methods

### 2.1 Study design

This cross-sectional study was conducted to assess the 12-month prevalence of work-related musculoskeletal disorders (WMSDs) and associated risk factors among older hospital cleaners in China. Data collection took place between August 25 and September 20, 2025 across three tertiary public hospitals in Wenzhou, eastern China: Wenzhou Central Hospital, the Second Affiliated Hospital of Wenzhou Medical University, and Ruian People’s Hospital. These facilities served as regional medical centers (each with > 1,000 hospital beds) and are representative of urban public hospitals in China.

The three hospitals employed approximately 500 cleaners in total, of whom approximately 375 (75%) were aged ≥ 50 years. Sample size was calculated based on a reported WMSDs prevalence of 70% among hospital cleaners (15, 50), with a precision of 5% and confidence level of 95%, yielding a minimum required sample of 323 participants. The final sample of 326 participants (246 aged ≥ 50 years) met statistical analysis requirements.

### 2.2 Participants and sampling

Convenience sampling was employed to recruit hospital cleaners aged ≥ 50 years as the primary study population, with an additional group of cleaners aged < 50 years included as an age comparison group to assess age-gradient effects.

Inclusion criteria were: (1) employment in hospital cleaning for at least 12 months to ensure adequate familiarity with job tasks; (2) ability to understand questionnaire content and provide informed consent; (3) voluntary participation with written informed consent.

Exclusion criteria included: (1) congenital spinal deformities, or musculoskeletal trauma or surgery within the past 6 months; (2) cognitive or language impairments that prevented questionnaire comprehension; and (3) refusal to participate or provide informed consent. A total of 326 hospital cleaners were enrolled, comprising 246 (75.5%) aged ≥ 50 years as the main study cohort and 80 (24.5%) aged < 50 years as the age comparison group.

Hospital cleaners in the three participating hospitals worked various shift patterns, including regular day shifts (typically 6:30–17:00), split shifts, and rotating schedules covering early morning to evening hours. Weekly working hours ranged from approximately 40 to over 60 h, with extended hours often occurring due to staff shortages or increased workload demands during peak periods.

### 2.3 Questionnaire survey

#### 2.3.1 WMSDs assessment

The WMSDs were assessed using the Chinese version of the Standardized Nordic Musculoskeletal Questionnaire (C-NMQ) (51), a validated instrument previously used in studies of electronics manufacturing workers and information technology workers in China (52–54).

The questionnaire comprised three sections: (1) demographic characteristics (gender, age, job tenure, BMI, smoking status, etc.); (2) musculoskeletal symptoms across nine body regions (neck, shoulder, upper back, elbow, lower back, wrist/hand, hip/thigh, knee, and calf/foot); and (3) work-related factors including awkward postures, lifting or carrying heavy objects, repetitive tasks, prolonged standing, and vibrating tool use. In total, 72 risk factor variables were coded according to established scales.

Binary variables were coded as 0 (no) or 1 (yes). Ordinal variables were coded hierarchically: for instance, frequency of work breaks (“once,” “twice,” “three times,” “more than three times”) was coded as 1, 2, 3, and 4, respectively; daily total rest time (“≤30 min,” “30 min-1 h,” “1–2 h,” “≥2 h”) was coded in the same manner.

Following National Institute for Occupational Safety and Health (NIOSH) criteria (53, 55), WMSDs were defined as self-reported pain, discomfort, or numbness lasting ≥1 week in at least one of the nine body regions during the past 12 months, with symptoms emerging or worsening after starting current employment.

#### 2.3.2 Pilot study

Data were collected via electronic questionnaire. Prior to formal data collection, a pilot test involving five older cleaners (not included in the final sample) was conducted to assess questionnaire comprehensibility and electronic platform usability. The pilot test indicated an average completion time of 15–20 min, with all participants able to complete the questionnaire independently without comprehension difficulties.

#### 2.3.3 Data collection procedure

Formal data collection was conducted by two trained research assistants during cleaners’ break times. The standardized protocol included: (1) explaining the study purpose, questionnaire content, and procedural details; (2) obtaining written informed consent; (3) guiding participants to complete the electronic questionnaire via smartphone; (4) remaining available to address questions without influencing responses; and (5) immediately reviewing completed questionnaires for completeness, with missing items addressed on-site.

### 2.4 Statistical analysis

All data were analyzed using R version 4.3.3 (R Foundation for Statistical Computing, Vienna, Austria). Two-sided tests were performed with statistical significance set at *p* < 0.05.

Descriptive statistics were used to summarize the demographic characteristics and work-related factors of older hospital cleaners (≥50 years). Participants were stratified into four age groups (<50, 50–59, 60–69, ≥70 years), with 12-month WMSDs prevalence and 95% confidence intervals (CIs) calculated for each stratum. The Cochran-Armitage trend test was employed to evaluate linear trends in WMSDs prevalence across age groups. Seven-day and 12-month prevalence rates with 95% confidence intervals (CIs) were calculated for each of the nine body regions (neck, shoulder, upper back, elbow, lower back, wrist/hand, hip/thigh, knee, and calf/foot).

A two-stage analytical strategy was employed to identify independent WMSDs risk factors. First, univariable analyses (chi-square or Fisher’s exact tests) were conducted for all 72 potential occupational risk factors, and variables with *p* < 0.05 were entered into multivariable modeling. Second, multivariable logistic regression with backward stepwise selection based on Akaike Information Criterion (AIC) was performed (56). At each step, the variable whose removal resulted in the greatest reduction in AIC was eliminated until minimum AIC was achieved. For the final model, Wald *χ^2^* test *p*-values, adjusted odds ratios (*AOR*s), and 95% CIs were reported. Model fit was assessed via Hosmer-Lemeshow goodness-of-fit test (57). Discriminative ability was evaluated using area under the receiver operating characteristic curve (AUC) (58), with AUC values of 0.7–0.8 considered acceptable, 0.8–0.9 excellent, and > 0.9 outstanding (58). At the default threshold of 0.5, sensitivity, specificity, positive predictive value (PPV), and negative predictive value (NPV) were calculated to further assess classification performance.

#### 2.4.1 Sensitivity analysis

Sensitivity analyses were conducted to assess result robustness. Based on the final multivariable model, we additionally adjusted for weekly working hours (≤50 vs. >50 h) and compared *AOR* changes before and after adjustment. Following Mickey and Greenland’s simulation study recommendations (59), results were considered robust when *AOR* changes were ≤ 15%. We also compared AIC and AUC values between the two models to evaluate changes in model performance after incorporating working hours.

## 3 Results

### 3.1 Demographic characteristics of participants (≥50 years, *n* = 246)

Age distribution was as follows: 159 (64.6%) aged 50–59 years, 80 (32.5%) aged 60–69 years, and 7 (2.8%) aged ≥ 70 years. Regarding occupational characteristics, job tenure distribution was: 72 (29.3%) with 1–2 years, 90 (36.6%) with 2–5 years, 56 (22.8%) with 5–10 years, and 28 (11.4%) with ≥ 10 years. Work type distribution was: 148 (60.2%) cleaning only, 44 (17.9%) delivery only, and 54 (22.0%) both cleaning and delivery. BMI distribution was: 3 (1.2%) underweight, 134 (54.5%) normal weight, 91 (37.0%) overweight, and 18 (7.3%) obese. Educational level was: 109 (44.3%) primary school or below, 113 (45.9%) junior high school, 19 (7.7%) high school, and 5 (2.0%) college or above. Working time characteristics revealed a mean weekly working time of 52.0 h (*SD* = 10.6), with 104 (42.3%) working > 50 h per week. Sixty-seven participants (27.2%) engaged in shift work (night shifts).

**Figure 1:**
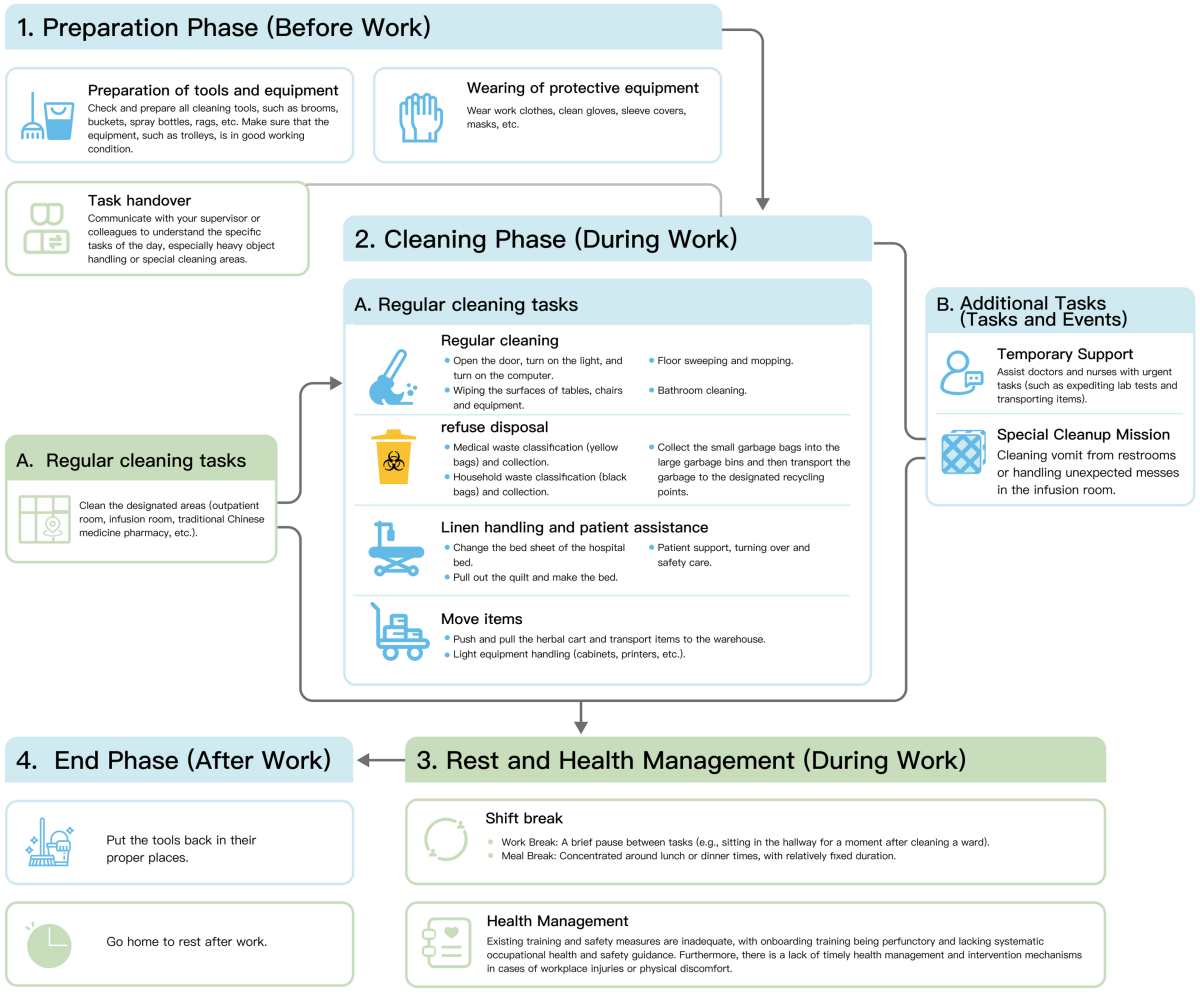
Daily cleaning tasks of hospital cleaners.

The analysis indicated that older hospital cleaners’ work was characterized by high physical demands and poor ergonomic conditions (Table 1). In terms of physical workload, 56.9% (140 participants) frequently lifted heavy objects (>5 kg per lift) and 39.4% (*n* = 97) lifted very heavy objects (>20 kg per lift). These lifting tasks reflect substantial physical burden. Additionally, 62.2% (*n* = 153) performed work requiring upper limb or hand force, such as repetitive wiping, lifting, carrying, and wringing operations. Furthermore, 28.0% (*n* = 69) used vibrating tools, and 24.8% (*n* = 61) drove cleaning vehicles. Nearly half of the cleaners (*n* = 121, 49.2%) reported performing multiple repetitive operations per minute (Table 1). Beyond regular cleaning duties, most cleaners undertook additional auxiliary tasks. These additional auxiliary tasks included operating department equipment, assisting healthcare staff with moving items, changing linens, transporting beds, and supporting patients. Some positions also required waste segregation and transportation, separating medical waste from general waste and transporting them to different designated locations (Figure 1). These additional responsibilities were particularly frequent during peak hours, further increasing the physical workload.

**Table 1:** Working postures and workload in older hospital cleaners (*n* = 246).

Working postures and workload	*n* (%)
Prolonged standing work	228 (92.70)
Prolonged sitting work	60 (24.40)
Prolonged squatting or kneeling work	76 (30.90)
Lifting heavy objects (>5 kilograms each time)	140 (56.90)
Lifting very heavy objects (>20 kilograms each time)	97 (39.40)
Work requiring upper limb/hand force	153 (62.20)
Using vibrating tools at work	69 (28.00)
Vehicle driving	61 (24.80)
Working in uncomfortable postures	57 (23.20)
Multiple repetitive operations per minute	121 (49.20)

For ergonomic conditions, prolonged standing was reported by 228 participants (92.7%), indicating that prolonged standing was the predominant working posture. Although the proportions reporting prolonged sitting (24.4%) and squatting/kneeling (30.9%) were relatively lower, these exposures remained notable. Another 23.2% (*n* = 57) frequently worked in uncomfortable postures, potentially involving high, low, or confined spaces.

Among demographic and work-related characteristics, weekly working hours showed a significant association with WMSDs prevalence (*p* < 0.001): prevalence was 50.0% (52/104) among those working > 50 h per week, significantly higher than 19.0% (27/142) among those working ≤ 50 h. Other characteristics (age, gender, job tenure, education level, BMI, work type, and shift work) showed no significant associations (Table 2).

**Table 2:** Demographic and occupational characteristics of older hospital cleaners (*n* = 246) and 12-month WMSDs prevalence.

Characteristic	Subgroup	Total (*n* = 246)	MSDs^1^	*p*
Age (year)	50–59	159 (64.6%)	54/159 (34%)	0.529ᵇ
	60–69	80 (32.5%)	24/80 (30%)	
	≥70	7 (2.9%)	1/7 (14.3%)	
Gender	Male	80 (32.5%)	24/80 (30%)	0.186
	Female	166 (67.5%)	55/166 (33.1%)	
Job tenure (years)	1–2	72 (29.3%)	29/72 (40.3)	0.186
	2–5	90 (36.6%)	24/90 (26.7%)	
	5–10	56 (22.8%)	15/56 (26.8%)	
	≥10	28 (11.4%)	11/28 (39.3%)	
Education level	Primary or below	109 (44.3%)	34/109 (31.2)	0.976^b^
	Junior high school	113 (45.9%)	37/113 (32.7)	
	High school	19 (7.7%)	6/19 (31.6)	
	College or above	5 (2%)	2/5 (40.0)	
Shift work	Yes	67 (27.2%)	20/67 (29.9)	0.755
	No	179 (72.8%)	59/179 (33.0)	
BMI	<18.5	3 (1.2%)	1/3 (33.3)	0.521ᵇ
	18.5–24.9	134 (54.5%)	38/134 (28.4)	
	25–29.9	91 (37%)	34/91 (37.4)	
	>30	18 (7.3%)	6/18 (33.3)	
Work type	Cleaning only	148 (60.2%)	50/148 (33.8)	0.788
	Delivery only	44 (17.9%)	13/44 (29.5)	
	Both	54 (22%)	16/54 (29.6)	
Weekly working hours	≤50 h	142 (57.7%)	27/142 (19.0)	<0.001*
	> 50 h	104 (42.3%)	52/104 (50.0)	

^1^MSDs in the past 12 months, *p < 0.05 (*p* from *χ*^2^ tests), 𝑿isher’s exact test when expected cell <5.

### 3.2 Overall and stratified WMSDs prevalence

A total of 246 hospital cleaners aged ≥ 50 years participated in this cross-sectional study. The 12-month prevalence of WMSDs was 32.1% (79/246, 95% CI: 26.6–38.2%). To further assess WMSDs patterns, we further examined prevalence across different age groups and body regions.

#### 3.2.1 Age-stratified prevalence (full sample, *N* = 326)

To examine how WMSDs prevalence varied across age groups, we analyzed the full sample including a younger comparison group (<50 years, *n* = 80). The 12-month WMSDs prevalence was 50.0% (40/80, 95% CI: 39.3–60.7%) among cleaners aged < 50 years, 34.0% (54/159, 95% CI: 27.1–41.6%) among those aged 50–59 years, 30.0% (24/80, 95% CI: 21.1–40.8%) among those aged 60–69 years, and 14.3% (1/7, 95% CI: 2.6–51.3%) among those aged ≥ 70 years.

**Figure 2:**
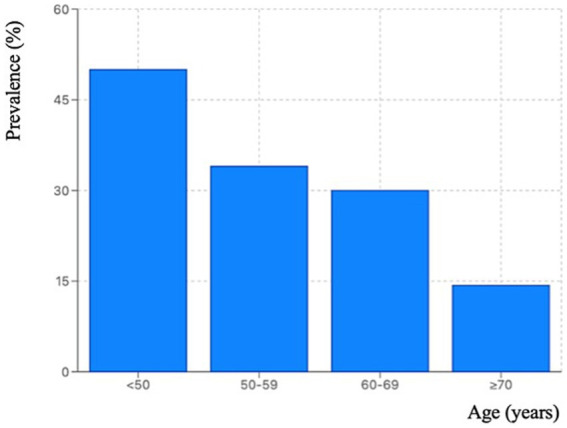
Age-stratified 12-month prevalence of WMSDs among hospital cleaners in Wenzhou, China (*n* = 326).

The Cochran-Armitage trend test revealed a statistically significant decline across age groups (*χ^2^* = 9.681, *p* = 0.021) (Figure 2). However, the extremely small sample size in the ≥70 age group (*n* = 7) and the wide confidence interval limit the reliability of this estimate. When the analysis was restricted to workers aged ≥ 50 years (excluding the younger comparison group), age-related differences were no longer statistically significant (*p* = 0.529, Table 2), likely due to reduced sample size and narrower age range.

#### 3.2.2 Body region-specific WMSD prevalence

Over the past 12 months, the most commonly affected body regions were shoulder (*n* = 36, 14.6%), knee (*n* = 31, 12.6%), neck (*n* = 24, 9.8%), calf/foot (*n* = 22, 8.9%), upper back (*n* = 20, 8.1%), lower back (*n* = 19, 7.7%), wrist/hand (*n* = 16, 6.5%), elbow (*n* = 13, 5.3%), and hip/thigh (*n* = 11, 4.5%).

Over the past 7 days, prevalence was highest for calf/foot (*n* = 16, 6.5%), followed by shoulder (*n* = 15, 6.1%), and neck, wrist/hand, and knee (*n* = 13, 5.3%) (Table 3). Shoulders, knees, and neck consistently showed high prevalence both over the past 12 months and the past 7 days.

**Table 3:** Multivariable logistic regression of WMSDs risk factors in older hospital cleaners (*n* = 246).

Body region	7-day prevalence *n* (%)	12-month prevalence *n* (%)
Overall	41 (16.7)	79 (32.1)
Neck	13 (5.3)	24 (9.8)
Shoulder	15 (6.1)	36 (14.6)
Upper back	12 (4.9)	20 (8.1)
Elbow	9 (3.7)	13 (5.3)
Lower back	11 (4.5)	19 (7.7)
Wrist/Hand	13 (5.3)	16 (6.5)
Hip/Thigh	9 (3.7)	11 (4.5)
Knee	13 (5.3)	31 (12.6)
Calf/ Foot	16 (6.5)	22 (8.9)
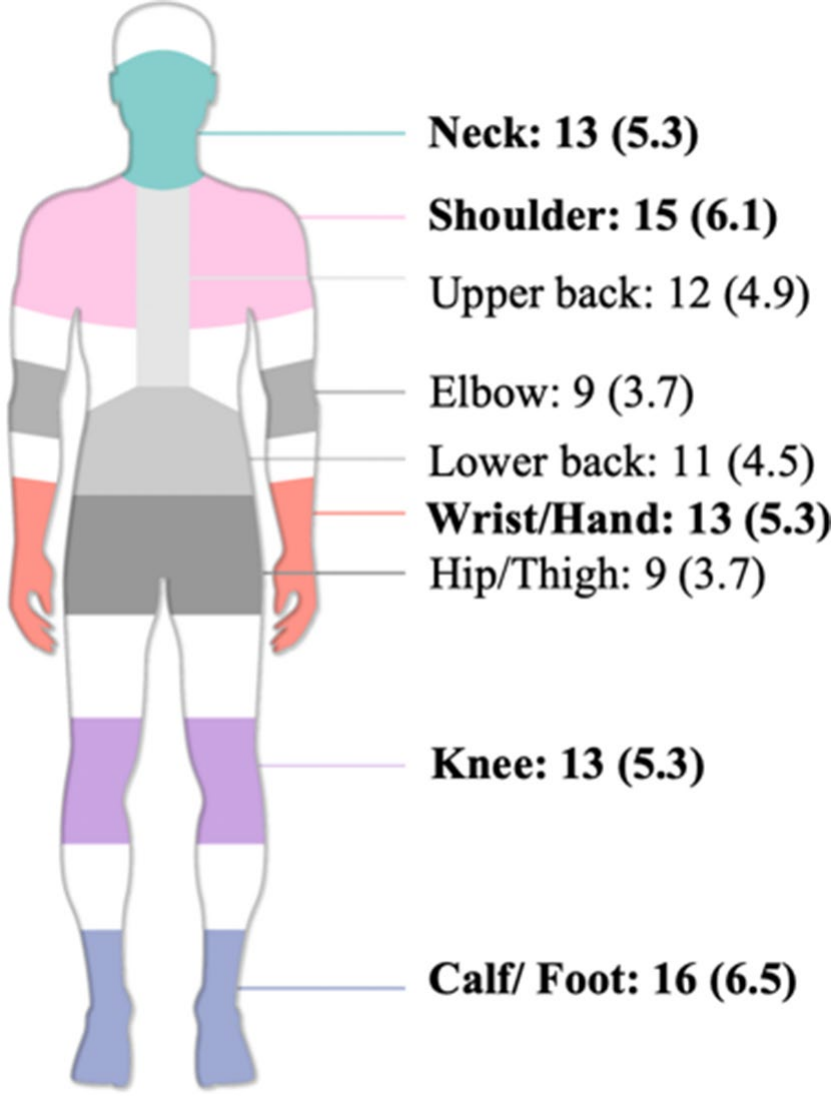		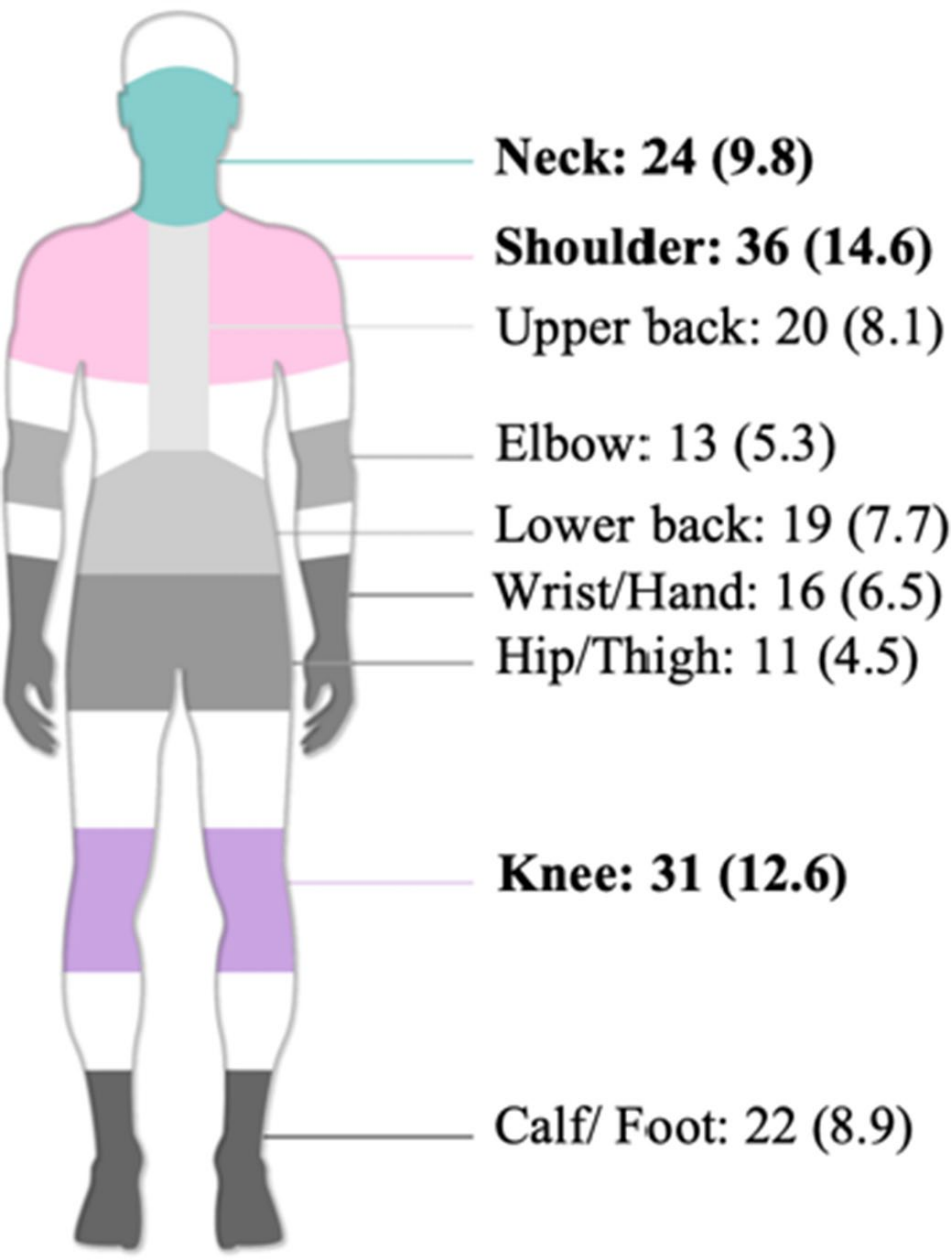

### 3.3 Risk factors associated with WMSDs

#### 3.3.1 Univariable analysis

In univariable analyses, chi-square tests (or Fisher’s exact tests when appropriate) were used to examine associations between potential occupational risk factors and WMSDs. Eighteen risk factors showed statistically significant associations and were included in multivariable analysis. These factors comprised awkward postures (working in uncomfortable postures, neck flexion, neck twisting, simultaneous bending and twisting, prolonged twisted postures, deep forward bending, deep twisting, prolonged forward-bent postures); physical loads (lifting heavy objects > 5 kg, lifting very heavy objects > 20 kg, work requiring upper limb/hand force, use of vibrating tools, frequent gripping of vibrating floor scrubbers); repetitive movements (multiple repetitive operations per minute, head performing same motion multiple times per minute, upper arms/fingers performing same motion multiple times per minute, trunk performing same motion multiple times per minute); prolonged sitting work; and organizational factors (staff shortage, reaching with hands or arms).

#### 3.3.2 Multivariable logistic regression analysis

Backward stepwise logistic regression identified four occupational risk factors in the main model (Table 4). After adjusting for other variables, two factors showed statistically significant association with WMSDs. Working in uncomfortable postures was the strongest risk factor (*AOR* = 2.87, 95% CI: 1.39–5.95, *p* = 0.004), indicating that exposed workers had 2.87 times the risk compared to unexposed workers. Staff shortage in department also showed significant association (*AOR* = 2.71, 95% CI: 1.38–5.32, *p* = 0.004), with cleaners in understaffed departments having 2.71 times the risk compared to those in adequately staffed departments.

**Table 4:** Multivariable logistic regression of WMSDs risk factors in older adult hospital cleaners (*n* = 246).

Risk factors	Partial regression coefficient (*B*)	Standard Error (*SE*)	Wald’s *χ^2^*	*p*	AOR	95%CI
Working in uncomfortable postures	1.055	0.368	8.19	0.004**	2.87	1.39–5.95
Neck flexion or prolonged maintenance of this posture	0.551	0.365	2.28	0.131	1.73	0.84–3.53
Staff shortage in department	0.996	0.342	8.47	0.004**	2.71	1.38–5.32
Use of vibrating tools	0.612	0.334	3.36	0.067	1.84	0.95–3.54

**p < 0.01; AOR, adjusted odds ratio; 95% CI, 95% confidence interval.

Use of vibrating tools (*B* = 0.612, *p* = 0.067) and neck flexion or prolonged maintenance of this posture (*B* = 0.551, *p* = 0.131) did not show a significant association with WMSDs prevalence among older hospital cleaners, although odds ratios suggested potential risk.

#### 3.3.3 Model performance and validation

The logistic regression model demonstrated good calibration. The Hosmer-Lemeshow test indicated satisfactory model fit (*χ^2^* = 2.69, *df* = 4, *p* = 0.611). There was no significant deviation between observed values and predicted probabilities. The model showed moderate discriminative ability, with an area under the ROC curve (AUC) of 0.722 (95% CI: 0.66–0.78). At the default threshold of 0.5, overall classification accuracy was 74.8%, with sensitivity of 65.5%, specificity of 77.5%, positive predictive value of 45.6%, and negative predictive value of 88.6%.

#### 3.3.4 Sensitivity analysis

Sensitivity analyses adjusting for weekly working hours confirmed the robustness of the two primary risk factors (Table 5). Uncomfortable postures (*AOR* = 3.22, 95% CI: 1.53–6.79, *p* = 0.002) and staff shortage (*AOR* = 2.62, 95% CI: 1.29–5.32, *p* = 0.007) remained significantly associated with WMSDs, with *AOR* changes of +12.2% and −3.3%, respectively. Both were below the 15% confounding threshold recommended by Mickey and Greenland (59). The association with vibrating tool use showed substantial attenuation (*AOR* change: −22.3%, from 1.84 to 1.43, *p* = 0.301), indicating potential confounding by working hours.

**Table 5:** Full results of sensitivity analysis models.

Variable	Model 1 (Main model)		Model 2 (+ Weekly working hours)	
	*B* (SE)	AOR (95% CI)	*B* (SE)	AOR (95% CI)
Intercept	−1.598 (0.222)	0.20***	−2.172 (0.286)	0.11***
Working in uncomfortable postures	1.055 (0.368)	2.87 (1.39–5.95)**	1.169 (0.386)	3.22 (1.53–6.79)**
Neck flexion/prolonged maintenance	0.551 (0.365)	1.73 (0.84–3.53)	0.444 (0.375)	1.56 (0.74–3.29)
Staff shortage	0.996 (0.342)	2.71 (1.38–5.32)**	0.963 (0.359)	2.62 (1.29–5.32)**
Use of vibrating tools	0.612 (0.334)	1.84 (0.95–3.54)	0.361 (0.348)	1.43 (0.72–2.86)
Weekly working hours >50 h	–	–	1.346 (0.317)	3.84 (2.05–7.18)***
AIC	280.38		263.70	
AUC	0.722		0.766	
Sample size	246		246	
AIC	280.38		263.70	

**p < 0.01, ***p < 0.001.

Weekly working hours > 50 h was identified as a strong independent risk factor (*A*OR = 3.84, 95% CI: 2.05–7.18, *p* < 0.001). Incorporating working hours improved model fit and discriminative ability.

## 4 Discussion

### 4.1 WMSDs prevalence

This study examined the prevalence of WMSDs among hospital cleaners aged ≥50 years in Wenzhou, China, and identified associated risk factors. We found a 12-month WMSDs prevalence of 32.1%. The prevalence is lower than rates reported in Ethiopia (57.2%) (20), Thailand (81.9%) (21), Texas (66.66%) (15), and the United Kingdom (74%) (50). Several factors may explain this variation. First, our study specifically focused on older cleaners aged ≥ 50 years who had remained employed, whereas most international studies did not stratify by age. Due to the healthy worker effect, individuals who are unable to tolerate musculoskeletal symptoms may withdraw from physically demanding cleaning tasks before reaching older age, leading to lower observed prevalence in older cleaners compared with all-age samples (44, 60). Second, differences in workplace environments and ergonomic practices may influence musculoskeletal risk. We observed that some participating hospitals had implemented preliminary ergonomic improvements, such as replacing traditional wringing mops with replaceable cloth-head mops that are centrally cleaned by logistics departments. This change may help reduce repetitive neck flexion and arm abduction motions (61). Previous research has demonstrated that ergonomic cleaning equipment can reduce musculoskeletal strain among cleaning workers (62). This may help explain why wrist/hand prevalence in our study (6.5%) was lower than rates reported in the United Kingdom (22%) (50), France (44%) (43), and Brazil (27.5%) (16). Third, self-reported questionnaire-based assessments without clinical confirmation may be influenced by recall errors and subjective reporting (63, 64). Moreover, economic dependence on work and concerns about job stability may contribute to underreporting of symptoms among older cleaners (12), the concern also observed in a Turkish study of cleaning workers (22).

Despite the comparatively lower prevalence observed in our sample, WMSDs remain a significant occupational health concern. Nearly one-third of older cleaners reported WMSD symptoms, and the prevalence reached 50.0% among those working more than 50 h per week. In the context of ongoing delayed retirement policies in China, these findings indicate a potentially escalating occupational health burden that requires timely and systematic prevention strategies and workplace interventions (65).

#### 4.1.1 Body region-specific prevalence and ergonomic explanations

Over the past 12 months, the shoulder (14.6%), knee (12.6%), neck (9.8%), and calf/foot (8.9%) were the most frequently affected body regions among older hospital cleaners. The high prevalence of shoulder symptoms aligns with findings reported in studies from Ethiopia and Norway (20, 66). Hospital cleaning involves repetitive overhead reaching and forceful upper-limb exertion, such as wiping high surfaces (e.g., walls, windows, equipment tops) and pushing or pulling cleaning equipment (e.g., floor scrubbers, cleaning carts) (67–70). These biomechanical demands increase the likelihood of subacromial impingement and rotator cuff tendinopathy (71). The finding that 62.2% of participants reported tasks requiring upper limb or hand force further exacerbates shoulder loading (Table 1).

The higher prevalence observed in the knee (12.6%) and calf/foot (8.9%) may be attributable to frequent squatting, kneeling, standing, and walking activities. Prolonged standing (reported by 92.7%) can impair lower-limb venous return and induce muscle fatigue (72). Additionally, 30.9% reported prolonged squatting or kneeling work (Table 1). The relatively low wrist/hand prevalence (6.5%) may reflect recent ergonomic improvements. For instance, some hospitals have replaced traditional wringing mops with removable cloth mop heads that are centrally cleaned by logistics departments, reducing repetitive wrist twisting motions.

However, the prevalence of neck (9.8%) and lower back (7.7%) symptoms remains noteworthy. Moreover, 23.2% of participants reported frequently working in uncomfortable postures, such as sustained neck flexion when wiping low surfaces, or trunk twisting and forward bending when carrying or mopping. Such movements increase biomechanical strain on the cervical and lumbar spine (16, 73, 74).

#### 4.1.2 Age-related WMSDs patterns

Among the full sample including the younger comparison group (<50 years), the WMSD prevalence decreased across age groups. This age gradient may reflect workforce dynamics under delayed retirement reforms. With the implementation of gradual delayed retirement policies beginning in China (75), increasing numbers of cleaners will need to continue working into older ages. The finding that younger cleaners exhibited the highest prevalence may indicate that WMSDs often begin to develop during midlife. Without timely prevention, these conditions may progressively worsen as working years are extended (39).

When the analysis was restricted to the older group (≥ 50 years), age differences were no longer statistically significant (*p* = 0.529), indicating that chronological age itself may not be the primary determinant of WMSDs within this population. The finding is consistent with a Brazilian study (76) and supports the healthy worker effect. As a result, work environment factors (e.g., postural demands, staffing levels) may become more influential determinants of WMSDs among those who remain employed.

It should be noted that the ≥ 70 years subgroup had a very small sample size (*n* = 7, 95% CI: 2.6–51.3%), resulting in wide confidence intervals and reduced estimate stability. Furthermore, because this was a cross-sectional study, we were unable to obtain health information on workers who had already left their positions. Thus, this limitation prevents direct verification of the healthy worker effect. Future research should employ longitudinal cohort designs that track workers from employment entry through job departure to better evaluate causal relationships among age, exposure duration, and musculoskeletal outcomes.

### 4.2 Risk factors for WMSDs

WMSDs are multifactorial conditions influenced by biomechanical, individual, psychosocial, and organizational factors (77, 78). In this study, multivariable logistic regression analysis identified two occupational factors independently associated with WMSDs among hospital cleaners aged ≥50 years: working in uncomfortable postures (*AOR* = 2.87) and staff shortage (*AOR* = 2.71). Sensitivity analyses further indicated that working more than 50 h per week was also a significant risk factor (*AOR* = 3.84), highlighting that excessive workload may further increase musculoskeletal strain in this population. Taken together, these results underscore the importance of addressing both biomechanical (e.g., awkward postures) and organizational (e.g., staffing adequacy, workload demands) determinants when designing prevention strategies for older hospital cleaners.

#### 4.2.1 Working in uncomfortable postures

The unique characteristics of hospital cleaning work necessitate frequent awkward postures, including: (1) combined trunk flexion and rotational movements (bending to mop, twisting to wipe); (2) prolonged upper-limb elevation (wiping high surfaces); (3) prolonged squatting or kneeling (cleaning low areas, corners); and (4) neck flexion or extension (cleaning under beds, ceilings) (42, 43, 79). From a biomechanical perspective, these awkward postures increase mechanical load on joints and surrounding soft tissues (80).

Notably, although most cleaners (92.7%) reported prolonged standing work, “prolonged standing” was not retained in the multivariable model, whereas “working in uncomfortable postures” remained significantly associated with WMSDs. This may indicate that posture quality (i.e., whether awkward postures are adopted) is more important than standing duration alone. This finding was confirmed by research conducted in the UK, Turkey, and Ethiopia (26, 50, 81). Therefore, optimizing work postures, rather than merely reducing standing time, may be more critical for preventing musculoskeletal strain among older cleaners.

#### 4.2.2 Staff shortage

Staff shortage was another significant risk factor. Understaffing means individual cleaners must cover larger cleaning areas and perform more tasks, with less rest time, which may increase both the duration and intensity of physical exposure (75). Moreover, time pressure and work intensity from understaffing may compound psychosocial stress and limit musculoskeletal recovery. Additionally, many outsourced cleaners face job insecurity, lower social status, and low wages. These conditions may exacerbate WMSD risk through psychosocial pathways. Evidence suggests that outsourcing key hospital services can produce adverse effects on population health, indicating a need for stronger oversight and labor protections (82). These findings highlight the need to improve staffing levels and labor protections to reduce WMSD risk among older cleaners.

#### 4.2.3 Weekly working hours > 50 hours

In the sensitivity analyses, working more than 50 h per week emerged as an independent risk factor (*AOR* = 3.84, 95% CI: 2.05–7.18), and incorporating weekly working hours improved model fit and discrimination ability (decreased AIC, increased AUC). This finding is consistent with existing evidence (83, 84). Failure to take breaks is associated with shoulder and knee pain (22). Prolonged physical labor may lead to poor circulation, fatigue accumulation, muscle tension, and insufficient recovery time, thereby elevating the risk of WMSDs, such as shoulder pain (22, 85–87).

#### 4.2.4 Other potential risk factors

“Use of vibrating tools” (*AOR* = 1.84, *p* = 0.067) and “neck flexion or prolonged maintenance of this posture” (*AOR* = 1.73, *p* = 0.131) did not reach statistical significance in the multivariable models, though the effect estimates suggested potential elevated risk. Sensitivity analyses showed that the association with vibrating tool use was substantially attenuated after incorporating working hours (*AOR* decreased from 1.84 to 1.43). One possible explanation is that cleaners who use vibrating tools may undertake more work tasks and therefore accumulate longer working hours and heavier workloads. Similar patterns have been reported in research conducted in the UK (61). These findings suggest that the apparent effect of vibrating tool use may be partly mediated by workload-related factors.

### 4.3 Implications for practice

#### 4.3.1 Ergonomic improvements

Given that working in uncomfortable postures was the strongest risk factor, cleaning tools and workflows should be ergonomically redesigned. Such improvements can reduce shoulder, neck, and knee loads among older hospital cleaners. For example, using lightweight and height-adjustable mops, dusters, spray bottles, knee pads, and non-slip tool handles may help reduce musculoskeletal strain (88). In addition, the use of transfer aids and lifting devices can minimize bending and heavy manual handling demands (25, 88). Assistive technologies and intelligent automation applications, such as cleaning robots, may further reduce manual workload and support sustainable work ability among aging cleaners (89). Importantly, involving cleaners in the selection and evaluation of cleaning equipment has been recommended to ensure that tools match task demands and user comfort (62). Furthermore, emerging technologies such as wearable sensor systems may enable more accurate real-time assessment and screening of WMSD risk, thereby supporting early intervention (90).

#### 4.3.2 Organizational management optimization

Addressing the significant risk factors of staff shortage (*AOR* = 2.71) and long working hours (*AOR* = 3.84), hospital management and outsourcing companies should ensure adequate staffing and keep weekly working hours within recommended limits. In addition, work task design should be optimized by distinguishing physically demanding tasks (e.g., carrying and transporting materials) from less physically intensive tasks (e.g., inspection and recording) to support age-friendly work environments. Encouraging alternating postures and establishing regular job rotation systems can further help minimize prolonged exposure to high-risk tasks (88).

#### 4.3.3 Occupational health training

To address the differing needs across the work lifespan, life-course-differentiated intervention strategies should be applied (91). For newly hired cleaners aged <50 years, priority should be given to occupational health surveillance with baseline WMSD screening, worker participation in work modifications, and early workplace-based intervention (92). For cleaners aged ≥50 years, regular health monitoring and training on musculoskeletal health awareness should be strengthened.

In addition, systematic and continuous occupational health training programs should be established, covering proper working postures, safe lifting techniques, and early recognition and reporting of WMSD symptoms. Current training practices are often one-off and insufficient, failing to cover all occupational risks (17). Therefore, ongoing, structured, and task-specific training is essential to effectively support musculoskeletal health among aging cleaners (93).

#### 4.3.4 Policy refinement

Older hospital cleaners typically have low educational attainment and are economically vulnerable, yet financial support systems for this group remain limited (81). To sustainably increase labor force participation among older workers, their health and well-being should be prioritized. In many low-income settings, hospital cleaners face inadequate policy enforcement, limited legal protections, and a lack of standardized procedures (83). In the context of delayed-retirement policies, WMSDs should be incorporated into priority occupational health screening programs. The management of outsourced labor should be standardized to ensure that outsourced cleaners receive equivalent occupational health protections. In addition, gradual retirement and flexible work arrangements may be explored to provide transitional options for older hospital cleaners who are unable to sustain full-time workloads.

### 4.4 Limitations

This study has several limitations. First, the cross-sectional design can only reveal associations, not causal relationships. The observed age gradient may reflect selection bias rather than a true protective effect of age. Future research should conduct longitudinal cohort studies tracking cleaners’ health trajectories from hiring to departure and should attempt to obtain health information from workers who have left their positions to directly verify the healthy worker effect. Second, self-reported data may introduce recall and reporting biases. WMSD determination without clinical examination or physician diagnosis may affect the accuracy of prevalence estimates. Additionally, differences in assessment tools, study populations, and national labor and welfare systems may limit the direct comparability of our results with those from other countries. Third, the ≥ 70-year group had a small sample size, limiting the stability of estimates for this stratum. Fourth, this study employed convenience sampling and was conducted only in Wenzhou, which may limit the generalizability of the findings. Finally, this study did not evaluate intervention effectiveness or cost–benefit. Future research should implement randomized controlled trials or quasi-experimental studies to assess the real-world impact of ergonomic improvements, organizational management optimization, and occupational health training interventions.

### 4.5 Future directions

Future research should employ longitudinal cohort designs to clarify causal relationships between work exposures and WMSDs across the work lifespan. Studies should also include workers who have left employment to directly assess the healthy worker effect. In addition, intervention-based research, such as randomized controlled trials or quasi-experimental studies, is needed to evaluate the effectiveness of ergonomic and organizational interventions. Furthermore, integrating wearable sensor technologies and digital risk assessment tools may support more precise exposure quantification and early detection of musculoskeletal strain. Finally, comparative research across regions and labor systems would help elucidate how organizational models and social welfare structures influence WMSD risks among hospital cleaners.

## 5 Conclusion

This study provides critical evidence on WMSDs among older hospital cleaners in China. This population has received insufficient attention despite constituting the majority of the hospital cleaning workforce. The findings indicate that without timely and targeted intervention, the health challenges experienced during middle age may intensify under delayed retirement policies, potentially compromising older workers’ ability to remain in employment.

The identification of key workplace-level determinants demonstrates that WMSDs in this population are driven primarily by modifiable occupational exposures, rather than aging alone. This shifts the focus of responsibility from individual workers to organizational and policy environments and underscores the need for employers and healthcare institutions to improve working conditions.

Our findings support a comprehensive prevention approach that aligns ergonomic, organizational, and occupational health measures. Improving task ergonomics, ensuring sufficient staffing, and regulating workload and working hours are central to reducing musculoskeletal strain and sustaining work ability among older cleaners. Establishing routine occupational health surveillance can further support early detection and timely intervention.

This study contributes evidence to inform the development of age-responsive labor protection and occupational health policymaking in the context of workforce aging and delayed retirement policies in China. Given the potential irreversibility of musculoskeletal impairment and the economic vulnerability of this workforce, the implications are both urgent and substantial (94, 95). Future research should incorporate multicenter longitudinal designs and evaluate intervention effectiveness to further guide sustainable and equitable employment conditions for aging workers in physically demanding occupations.

## Data Availability

The raw data supporting the conclusions of this article will be made available by the authors, without undue reservation.

## References

[1] Silverstein M. Meeting the challenges of an aging workforce. Am J Ind Med. (2008) 51:269–80. doi: 10.1002/ajim.20569, PMID: 18271000

[2] Bardy R. Pre-retirement, retirement, and post-retirement: policy considerations and consequences. Public Adm Policy. (2025) 28:8–18. doi: 10.1108/PAP-03-2024-0034

[3] Vitrano G Micheli GJ. Rethinking work in industry 5.0: leveraging technology for an ageing workforce. Public Health Challenges. (2025) 4:e70130. doi: 10.1002/puh2.70130, PMID: 40994934 PMC12455513

[4] Goderis B Muns S. Decent old-age incomes for all? A microdata analysis of poverty among older adults in the Netherlands. Int J Soc Welfare. (2025) 34:e70020. doi: 10.1111/ijsw.70020

[5] Huang J. The impact of delayed retirement age on workers' physical health: a survey and discussion based on Chinese workers. In: Chew FP editor. Global dialogue on media dynamics, trends and perspectives on public relations and communication. Boca Raton, FL: CRC Press (2025). 235–9.

[6] Guan G-f Wang L Ma D-f Luo P-s. Research on the impact of delayed retirement on the subjective wellbeing of older adults. Front Public Health. (2025) 13:1530613. doi: 10.3389/fpubh.2025.1530613, PMID: 40556928 PMC12185451

[7] The Organization for Economic Cooperation and Development. Labour force participation rate. (2025). Available online at: https://www.oecd.org/en/data/indicators/labour-force-participation-rate.html (Accessed November 3, 2025).

[8] Hofäcker D Naumann E. The emerging trend of work beyond retirement age in Germany. Z Gerontol Geriatr. (2015) 48:473–9. doi: 10.1007/s00391-014-0669-y, PMID: 25117859

[9] Yeo H. Are there sufficient jobs for older workers in a local market? Educ Gerontol. (2025) 51:1083–100. doi: 10.1080/03601277.2025.2450135

[10] Demirel S Ozdemir S. Development of the hospital hygiene standard precautions scale: cleaning staff version. Int J Occup Saf Ergon. (2025) 31:468–77. doi: 10.1080/10803548.2025.2452732, PMID: 39957569

[11] Cetrulo A Manicardi C Moro A. Automation in cleaning: why dirty, invisible, and risky jobs will not be replaced by robots yet. In: Cirillo V Rinaldini M Virgillito ME editors. Technology and work in services: Vulnerable workers under automation and digitalisation. Cham: Springer (2025) 97–129.

[12] Burkhalter D Wagner A Feer S Wieber F Ihle A Baumann I. Financial reasons for working beyond the statutory retirement age: risk factors and associations with health in late life. Int J Environ Res Public Health. (2022) 19:10505. doi: 10.3390/ijerph191710505, PMID: 36078221 PMC9518211

[13] Oakman J Clays E Jørgensen MB Holtermann A. Are occupational physical activities tailored to the age of cleaners and manufacturing workers? Int Arch Occup Environ Health. (2019) 92:185–93. doi: 10.1007/s00420-018-1364-x, PMID: 30374698

[14] Park J Kim SG Park J-s Han B Kim KB Kim Y. Hazards and health problems in occupations dominated by aged workers in South Korea. Ann Occup Environ Med. (2017) 29:27. doi: 10.1186/s40557-017-0177-9 28670457 PMC5485698

[15] Salwe K Kumar S Hood J. Nonfatal occupational injury rates and musculoskeletal symptoms among housekeeping employees of a hospital in Texas. J Environ Public Health. (2011) 2011:382510. doi: 10.1155/2011/382510, PMID: 21776437 PMC3136138

[16] Luz EMF Munhoz OL Greco PBT Morais BX Camponogara S Magnago TSBS. Prevalence and factors associated with musculoskeletal pain among hospital cleaning staff. Rev Bras Enferm. (2024) 77:e20230237. doi: 10.1590/0034-7167-2023-0237, PMID: 39699353 PMC11654551

[17] Cross S Gon G Morrison E Afsana K Ali SM Manjang T . An invisible workforce: the neglected role of cleaners in patient safety on maternity units. Glob Health Action. (2019) 12:1480085. doi: 10.1080/16549716.2018.1480085, PMID: 31154993 PMC6338282

[18] Akyildiz C. Proactive approaches to ensuring occupational health and safety of disadvantaged workers. In: Özsungur F Bekar F editors. Empowering employee proactive behavior: Concepts and perspectives from organization and society. Cham: Springer (2025) 67–96.

[19] Lin J-H Lee W Smith CK Yragui NL Foley M Shin G. Cleaning in the 21st century: the musculoskeletal disorders associated with the centuries-old occupation–a literature review. Appl Ergon. (2022) 105:103839. doi: 10.1016/j.apergo.2022.103839, PMID: 35809429

[20] Afework A Tamene A Tafa A. Musculoskeletal disorders and its associated factors among hospital cleaners in Addis Ababa, Ethiopia. Sci Rep. (2024) 14:2887. doi: 10.1038/s41598-024-53531-0, PMID: 38311673 PMC10838922

[21] Laithaisong T Aekplakorn W Suriyawongpaisal P Tupthai C Wongrathanandha C. The prevalence and risk factors of musculoskeletal disorders among subcontracted hospital cleaners in Thailand. J Health Res. (2022) 36:802–12. doi: 10.1108/JHR-01-2021-0040

[22] Medeni V Medeni İ Tosun M Uğraş Dikmen A İlhan M. Working conditions, health status, and musculoskeletal disorders among hospital cleaning workers: a cross-sectional study in Turkey. Med Pr. (2024) 75:397–413. doi: 10.13075/mp.5893.01509 39540699

[23] Punnett L Wegman DH. Work-related musculoskeletal disorders: the epidemiologic evidence and the debate. J Electromyogr Kinesiol. (2004) 14:13–23. doi: 10.1016/j.jelekin.2003.09.015, PMID: 14759746

[24] Roberts S Colombier P Sowman A Mennan C Rölfing JH Guicheux J . Ageing in the musculoskeletal system: cellular function and dysfunction throughout life. Acta Orthop. (2016) 87:15–25. doi: 10.1080/17453674.2016.1244750, PMID: 27748151 PMC5389428

[25] Okati-Aliabad H Habybabady RH. Work-related muscluskeletal disorders and work ability among hospital employees in southeast of Iran. Health Scope. (2024) 13:8901. doi: 10.5812/healthscope-148901

[26] Fentanew M Jember G Takele M Cherkos K Mamaye Y Zemariam A. Epidemiology and risk factors of low back pain among hospital cleaners in resource limited settings: a multi-centered cross-sectional study. Int J Clin Med Educ Res. (2023) 2:216–25.

[27] Yassi A. Work-related musculoskeletal disorders. Curr Opin Rheumatol. (2000) 12:124–30. doi: 10.1097/00002281-200003000-00006, PMID: 10751015

[28] Amell T Kumar S. Work-related musculoskeletal disorders: design as a prevention strategy. A review. J Occup Rehabil. (2001) 11:255–65. doi: 10.1023/A:1013344508217, PMID: 11826726

[29] Sebbag E Felten R Sagez F Sibilia J Devilliers H Arnaud L. The world-wide burden of musculoskeletal diseases: a systematic analysis of the World Health Organization burden of diseases database. Ann Rheum Dis. (2019) 78:844–8. doi: 10.1136/annrheumdis-2019-215142, PMID: 30987966

[30] Jia N Zhang H Ling R Liu Y Li G Ren Z . Epidemiological data of work-related musculoskeletal disorders—China, 2018–2020. China CDC weekly. (2021) 3:383–9. doi: 10.46234/ccdcw2021.104, PMID: 34594889 PMC8422204

[31] Dong S Yu Z Zhang S Li J. Multidimensional poverty dynamics and health among middle-aged and elderly people: a longitudinal study in China. Humanit Soc Sci Commun. (2025) 12:1–10. doi: 10.1057/s41599-025-05287-9

[32] Zhou L Zhu C Walsh CA Zhang X. Assessing the effect of health status on multidimensional poverty among older adults: the Chinese longitudinal healthy longevity survey. Front Public Health. (2023) 11:1150344. doi: 10.3389/fpubh.2023.1150344, PMID: 37475773 PMC10355057

[33] Man H Vinstrup J Andersen LL. Work and lifestyle factors associated with musculoskeletal pain among professional cleaners: a cross-sectional study. Int J Occup Saf Ergon. (2025) 31:522–8. doi: 10.1080/10803548.2025.2453319, PMID: 39936832

[34] Hacker CE Debono D Travaglia J Carter DJ. Falling through the cracks: the invisible hospital cleaning workforce. J Health Organ Manag. (2022) 36:981–6. doi: 10.1108/JHOM-02-2022-0035, PMID: 36116792

[35] Dancer SJ. Hospital cleaning in the 21st century. Eur J Clin Microbiol Infect Dis. (2011) 30:1473–81. doi: 10.1007/s10096-011-1250-x, PMID: 21499954

[36] Anderson SP Oakman J. Allied health professionals and work-related musculoskeletal disorders: a systematic review. Saf Health Work. (2016) 7:259–67. doi: 10.1016/j.shaw.2016.04.001, PMID: 27924228 PMC5127976

[37] Zhou J Xie S Xu S Zhang Y Li Y Sun Q . From pain to progress: comprehensive analysis of musculoskeletal disorders worldwide. J Pain Res. (2024) 17:3455–72. doi: 10.2147/JPR.S488133, PMID: 39469334 PMC11514690

[38] Shah D. Healthy worker effect phenomenon. Indian J Occupat Environ Med. (2009) 13:77–9. doi: 10.4103/0019-5278.55123, PMID: 20386623 PMC2847330

[39] Mozafari A Vahedian M Mohebi S Najafi M. Work-related musculoskeletal disorders in truck drivers and official workers. Acta Med Iran. (2015) 53:432–8. 26520631

[40] Zamora-Chávez SC Vásquez-Alva R Luna-Muñoz C Carvajal-Villamizar LL. Factores asociados a trastornos musculoesqueléticos en trabajadores de limpieza del servicio de emergencia de un hospital terciario. Rev Fac Med Hum. (2020) 20:388–96. doi: 10.25176/rfmh.v20i3.3055

[41] Gillen M Yen IH Trupin L Swig L Rugulies R Mullen K . The association of socioeconomic status and psychosocial and physical workplace factors with musculoskeletal injury in hospital workers. Am J Ind Med. (2007) 50:245–60. doi: 10.1002/ajim.20429, PMID: 17311255

[42] Schwartz A Gerberich SG Kim H Ryan AD Church TR Albin TJ . Janitor ergonomics and injuries in the safe workload ergonomic exposure project (SWEEP) study. Appl Ergon. (2019) 81:102874. doi: 10.1016/j.apergo.2019.102874, PMID: 31422267

[43] Koskas D Vignais N. Physical ergonomic assessment in cleaning hospital operating rooms based on inertial measurement units. Bioengineering. (2024) 11:154. doi: 10.3390/bioengineering11020154, PMID: 38391640 PMC10886191

[44] Xu H Zhang Z Yang X Yang Q Chen T. Effects of extended working lives on depressive symptoms, physical, and cognitive health in middle and later life: evidence from China. Soc Sci Med. (2025) 369:117833. doi: 10.1016/j.socscimed.2025.117833, PMID: 39955817

[45] Flaherty JH Liu ML Ding L Dong B Ding Q Li X . China: the aging giant. J Am Geriatr Soc. (2007) 55:1295–300. doi: 10.1111/j.1532-5415.2007.01273.x, PMID: 17661972

[46] So BCL Lee EWF Ng S Man SS. Prevalence and associated factors of work-related musculoskeletal disorder symptoms amongst emergency medical service workers. Sci Rep. (2025) 15:19806. doi: 10.1038/s41598-025-04945-x, PMID: 40473716 PMC12141473

[47] Liu F Duan Y Wang Z Ling R Xu Q Sun J . Mixed adverse ergonomic factors exposure in relation to work-related musculoskeletal disorders: a multicenter cross-sectional study of Chinese medical personnel. Sci Rep. (2025) 15:14705–12. doi: 10.1038/s41598-025-99477-9, PMID: 40289235 PMC12034785

[48] Smith DR Wei N Zhao L Wang R-S. Musculoskeletal complaints and psychosocial risk factors among Chinese hospital nurses. Occup Med. (2004) 54:579–82. doi: 10.1093/occmed/kqh117, PMID: 15576874

[49] Ilesanmi O Omotoso B Amenkhienan I. Accidents, injuries and the use of personal protective equipment, among hospital cleaners in a tertiary hospital in south West Nigeria. Res J Health Sci. (2015) 3:275–84. doi: 10.4314/rejhs.v3i4

[50] Woods V Buckle P. Musculoskeletal ill health amongst cleaners and recommendations for work organisational change. Int J Ind Ergon. (2006) 36:61–72. doi: 10.1016/j.ergon.2005.08.001

[51] Kuorinka I Jonsson B Kilbom A Vinterberg H Biering-Sørensen F Andersson G . Standardised Nordic questionnaires for the analysis of musculoskeletal symptoms. Appl Ergon. (1987) 18:233–7. doi: 10.1016/0003-6870(87)90010-X, PMID: 15676628

[52] Zhang D Lu L Hu H He Z Lin X Jia N . Analysis of risk factors of multi-site work-related musculoskeletal disorders among workers in the industry of electronic equipment manufacturing. China Occup Med. (2020) 6:253–9.

[53] Yang F Di N Guo W-w Ding W-b Jia N Zhang H . The prevalence and risk factors of work related musculoskeletal disorders among electronics manufacturing workers: a cross-sectional analytical study in China. BMC Public Health. (2023) 23:10. doi: 10.1186/s12889-022-14952-6, PMID: 36597111 PMC9809125

[54] Zheng B Chen F Wang J Deng H Li J Zhou C . The prevalence and correlated factors of occupational stress, cumulative fatigue, and musculoskeletal disorders among information technology workers: a cross-sectional study in Chongqing, China. Healthcare. (2023) 11:2322. doi: 10.3390/healthcare11162322, PMID: 37628520 PMC10454031

[55] Salvendy G. Handbook of human factors and ergonomics. New Jersey: John Wiley & Sons (2012).

[56] Akaike H. A new look at the statistical model identification. IEEE Trans Automat Control. (1974) 19:716–23. doi: 10.1109/TAC.1974.1100705

[57] Hosmer DW Lemesbow S. Goodness of fit tests for the multiple logistic regression model. Commun Stat-Theor Meth. (1980) 9:1043–69. doi: 10.1080/03610928008827941

[58] Mandrekar JN. Receiver operating characteristic curve in diagnostic test assessment. J Thorac Oncol. (2010) 5:1315–6. doi: 10.1097/JTO.0b013e3181ec173d, PMID: 20736804

[59] Mickey RM Greenland S. The impact of confounder selection criteria on effect estimation. Am J Epidemiol. (1989) 129:125–37. doi: 10.1093/oxfordjournals.aje.a115101, PMID: 2910056

[60] Börsch-Supan A Brugiavini A Croda E. The role of institutions and health in European patterns of work and retirement. J Eur Soc Policy. (2009) 19:341–58. doi: 10.1177/1350506809341515, PMID: 20428466 PMC2860194

[61] Woods V Buckle P. An investigation into the design and use of workplace cleaning equipment. Int J Ind Ergon. (2005) 35:247–66. doi: 10.1016/j.ergon.2004.09.004

[62] Chang JH Wu JD Liu CY Hsu DJ. Prevalence of musculoskeletal disorders and ergonomic assessments of cleaners. Am J Ind Med. (2012) 55:593–604. doi: 10.1002/ajim.22064, PMID: 22544565

[63] Bracciale A Manso MC Bracciale F Gavinha Costa L. Prevalence of self-reported musculoskeletal disorders in dentists—a cross-sectional study in Portugal and Italy. Healthcare. (2025) 13:1020. doi: 10.3390/healthcare13091020, PMID: 40361797 PMC12072067

[64] Zinabu FS Belay GJ Cherkos K Shiferaw KB Hailemariam TT Fentanew M . Prevalence and associated factors of musculoskeletal disorders among older adults living in Bahir Dar City Ethiopia cross-sectional study. Sci Rep. (2025) 15:37141. doi: 10.1038/s41598-025-21026-1, PMID: 41131106 PMC12549807

[65] Wahlström V Abtahi F Forsman M Yang L Öhrner P Tornevi A . Cardiovascular load and physical capacity in older workers engaged in physically demanding occupations. Int Arch Occup Environ Health. (2025) 98:673–83. doi: 10.1007/s00420-025-02161-8, PMID: 40810743 PMC12484335

[66] Lasrado OE Møllerløkken OJ Moen BE Van den Bergh G. Musculoskeletal symptoms among hospital cleaners. Arch Environ Occup Health. (2017) 72:87–92. doi: 10.1080/19338244.2016.1160862, PMID: 26954259

[67] Alford T Kempfert D. Scapular mobilization combined with thoracic manipulation for treating subacromial impingement syndrome in an elderly female: a case report. (2018). Available online at: https://soar.usa.edu/flsaspring2018/15 (Accessed November 3, 2025).

[68] De YTJ Tan AHC. Shoulder impingement syndrome, a common affliction of the shoulder: a comprehensive review. Proc Singapore Healthc. (2014) 23:297–305. doi: 10.1177/201010581402300406

[69] Burgel BJ White MC Gillen M Krause N. Psychosocial work factors and shoulder pain in hotel room cleaners. Am J Ind Med. (2010) 53:743–56. doi: 10.1002/ajim.20832, PMID: 20340100

[70] Rathod SS Desai DS. Prevalence of musculoskeletal disorders and its association with risk factors among cleaners of Vadodara: a cross-sectional study. J Soc Indian Physiother. (2023) 7:44–8. doi: 10.4103/jsip.jsip_29_23

[71] Lang AE Friesen KB Lawson J Mondal P Koehncke N Kim SY . Biomechanical risk factors for rotator cuff syndrome in high-risk occupations: a prospective study protocol. PLoS One. (2025) 20:e0326229. doi: 10.1371/journal.pone.0326229, PMID: 40540472 PMC12180629

[72] Coenen P Parry S Willenberg L Shi JW Romero L Blackwood DM . Associations of prolonged standing with musculoskeletal symptoms—a systematic review of laboratory studies. Gait Posture. (2017) 58:310–8. doi: 10.1016/j.gaitpost.2017.08.024, PMID: 28863296

[73] Madeleine P Søgaard K Holtermann A Samani A. Level of self-reported neck/shoulder pain and biomechanical workload in cleaners. Work. (2012) 41:447–52. doi: 10.3233/WOR-2012-0195-447, PMID: 22316765

[74] Boulais D Lal S Sztynda T Zaslawski C. Preserving lumbar spine physiology in the cleaning industry. J Health Saf Environ. (2017) 33:1–8.

[75] State Council of the People’s Republic of China. Interim measures for the implementation of the flexible retirement system (2024). Available online at: https://www.gov.cn/zhengce/zhengceku/202501/content_6995747.htm (Accessed November 3, 2025).

[76] Luz EMF Magnago TSBS Greco PBT Ongaro JD Lanes TC Lemos JC. Prevalence and factors associated with musculoskeletal pain in hospital cleaning workers. Texto Contexto-Enferm. (2017) 26:e00870016. doi: 10.1590/0104-07072017000870016

[77] Darvishi E Ghasemi F Sadeghi F Abedi K Rahmati S Sadeghzade G. Risk assessment of the work-related musculoskeletal disorders based on individual characteristics using path analysis models. BMC Musculoskelet Disord. (2022) 23:616. doi: 10.1186/s12891-022-05573-6, PMID: 35761242 PMC9235182

[78] Darvishi E Osmani H Aghaei A Moloud EA. Hidden risk factors and the mediating role of sleep in work-related musculoskeletal discomforts. BMC Musculoskelet Disord. (2024) 25:256. doi: 10.1186/s12891-024-07387-0, PMID: 38566113 PMC10985854

[79] Bello A Quinn MM Perry MJ Milton DK. Characterization of occupational exposures to cleaning products used for common cleaning tasks-a pilot study of hospital cleaners. Environ Health. (2009) 8:11. doi: 10.1186/1476-069X-8-11, PMID: 19327131 PMC2678109

[80] Santos T Cardoso VF Triches MI Gonçalves JS Moriguchi CS Sato TO. Is shoulder posture during work related to neck and shoulder symptoms among cleaners? Fisioter Mov. (2025) 38:e38107. doi: 10.1590/fm.2025.38107

[81] Genç A Kahraman T Göz E. The prevalence differences of musculoskeletal problems and related physical workload among hospital staff. J Back Musculoskelet Rehabil. (2016) 29:541–7. doi: 10.3233/BMR-160655, PMID: 26836838

[82] Goodair B Reeves A. The case against outsourcing from healthcare services. Gac Sanit. (2025) 38:102362. doi: 10.1016/j.gaceta.2024.102362 38309252

[83] Tolera ST Assefa N Gobena T Geremew A. Co-occurrence of occupational outcomes and associated factors among hospitals cleaners, eastern Ethiopia: a cross sectional study. BMC Public Health. (2024) 24:3108. doi: 10.1186/s12889-024-20571-0, PMID: 39529077 PMC11552356

[84] Melese H Gebreyesus T Alamer A Berhe A. Prevalence and associated factors of musculoskeletal disorders among cleaners working at Mekelle university, Ethiopia. J Pain Res. (2020) 13:2239–46. doi: 10.2147/JPR.S263319, PMID: 32982386 PMC7490036

[85] Hamberg-van Reenen HH Ariëns GA Blatter BM Van Der Beek AJ Twisk JW Van Mechelen W . Is an imbalance between physical capacity and exposure to work-related physical factors associated with low-back, neck or shoulder pain? Scand J Work Environ Health. (2006) 32:190–7. doi: 10.5271/sjweh.998 16804621

[86] Tolera ST Gobena T Geremew A Toseva E Assefa N. Work-related musculoskeletal disorders and associated factors among hospital sanitary workers in public hospitals of eastern Ethiopia. BMC Musculoskelet Disord. (2025) 26:640. doi: 10.1186/s12891-025-08873-9, PMID: 40616057 PMC12226844

[87] Kang YS Song CW. The pain of the shoulder joint and posterolateral area of upper arm. J Korean Pain Soc. (1996) 9:105–8.

[88] Padilla R Baltich J Fathallah F. Development of an ergonomic waste container for hospitals. Proceedings of the human factors and ergonomics society annual meeting. Los Angeles, CA: SAGE Publications Sage CA (2008).

[89] Megalingam RK Vadivel SRR Kotaprolu SS Nithul B Kumar DV Rudravaram G. Cleaning robots: a review of sensor technologies and intelligent control strategies for cleaning. J Field Robotics. (2025) 42:2234–59. doi: 10.1002/rob.22515

[90] Babangida AA Caraballo-Arias Y Decataldo F Violante FS. Advancing occupational medicine through wearable technology: a review of sensor Systems for Biomechanical Risk Assessment and Work-Related Musculoskeletal Disorder Prevention. ACS Sensors. (2025) 10:5410–32. doi: 10.1021/acssensors.5c01578, PMID: 40673699 PMC12379187

[91] Wagner C Carmeli C Jackisch J Kivimäki M van der Linden BW Cullati S . Life course epidemiology and public health. Lancet Public Health. (2024) 9:e261–9. doi: 10.1016/S2468-2667(24)00018-5, PMID: 38553145

[92] Sepehrian R Hashjin AA Farahmandnia H. A systematic review of programs and interventions for reduction of sickness absence in nursing staff with work-related musculoskeletal disorders. J Educ Health Promot. (2024) 13:205. doi: 10.4103/jehp.jehp_722_23, PMID: 39297114 PMC11410163

[93] de Souza Souza R. Occupational diseases of workers cleaning service in hospital environment: educational proposal to minimize exposure. Enferm Global. (2016) 15:552–64. doi: 10.1016/j.ajic.2015.01.029

[94] Tiwari J Halder P Sharma D Saini UC Rajagopal V Kiran T. Prevalence and association of musculoskeletal disorders with various risk factors among older Indian adults: insights from a nationally representative survey. PLoS One. (2024) 19:e0299415. doi: 10.1371/journal.pone.0299415, PMID: 39441775 PMC11498719

[95] Zaidi A. Life cycle transitions and vulnerabilities in old age: A review. USA: UNDP Human Development Report Office (2014).

